# Free Tissue Transfer in Sickle Cell Disease: A Case Report and Systematic Review

**DOI:** 10.1055/s-0043-1763260

**Published:** 2023-05-29

**Authors:** Anne Huang, Ronak A. Patel, Lawrence J. Gottlieb

**Affiliations:** 1 Section of Plastic and Reconstructive Surgery, University of Chicago Medicine and Biological Sciences, Chicago, Illinois

**Keywords:** free tissue transfer, free flap reconstruction, microsurgery, sickle cell disease

## Abstract

Hemoglobinopathies such as sickle cell disease (SCD) are traditionally considered a relative contraindication to free tissue transfer, due to concerns that erythrocyte sickling will increase the risk of microvascular thrombosis and flap failure. This article describes a case report with the successful use of free tissue transfer in a patient with SCD and provides a systematic literature review on free tissue transfer in SCD. A retrospective chart review was performed of a patient with SCD who underwent free tissue transfer at the authors' institution. A systematic literature review using the Preferred Reporting Items for Systematic Reviews and Meta-Analyses guidelines was performed using the keywords “free tissue transfer,” “free flap,” or “microsurgery” and “sickle cell” on PubMed, Ovid/Medline, and Scopus. A 29-year-old male with delayed presentation of an electrical burn to the face and scalp underwent wound closure with a free anterolateral thigh flap. Key management principles included red blood cell transfusion to keep hemoglobin S under 30% and hemoglobin greater than 10 g/dL, maintenance of hydration, normothermia, adequate analgesia, and postoperative anticoagulation. Systematic literature review identified 7 articles describing 13 cases of free tissue transfer in 10 patients with SCD, with combined complete free flap success in 10 of the 13 flaps. Free tissue transfer can be successfully performed in patients with SCD. However, evidence on the optimal management of this unique patient population in the perioperative period after free tissue transfer is limited to case reports in the literature.

## Introduction

Sickle cell disease (SCD) is an inherited hemoglobinopathy affecting around 1 in 500 African Americans, with an additional 1 in 12 being carriers of the autosomal recessive mutation. 1 Internationally, the prevalence of SCD is high among the sub-Saharan African, South Asian, Middle Eastern, and Mediterranean populations. With increasing human migration, the number of people affected by SCD is predicted to increase exponentially particularly in countries not historically endemic for SCD. 2

SCD was first described by James Herrick in 1910, who published a case report of severe anemia in a 20-year-old African male from Grenada with a “large number of thin, elongated, sickle-shaped, and crescent-shaped” erythrocytes. 3 Though Herrick did not understand the pathophysiology at that time, he astutely noted that “some unrecognized change in the composition of the corpuscle itself may be the determining factor.” In 1949, Pauling et al demonstrated that SCD was a “molecular disease” with a genetic basis for the abnormal hemoglobin S (HbS) protein. 4 HbS has single amino acid change from glutamine to valine in the β-globin chain of hemoglobin, causing polymerization in relative hypoxemia. SCD may be caused by the homozygous genotype HbSS, or it may be caused by compound heterozygous genotypes of HbS and other β-globin variants affecting structure or quantity, such as HbC and Hb β-thalassemia, respectively. Polymerization of HbS leads to changes in the structure and function of erythrocytes, including deformation into a sickle shape, increased stiffness, and increased adhesion. Repeated erythrocyte sickling shortens their lifespan, leading to chronic hemolytic anemia, microvascular thrombosis affecting all organs, and vaso-occlusive events. Furthermore, these events trigger a complex cascade leading to a proinflammatory and hypercoagulable state. 1 Known triggers of sickling include hypoxia, infection, dehydration, cold, circulatory stasis, and stress.

Traditionally, SCD is a relative contraindication for free tissue transfer, as obligate transient flap ischemia and tissue hypoxia may trigger sickling and microvascular thrombosis. Moreover, SCD patients are hypercoagulable and have increased risks of a wide range of perioperative and postoperative complications, including acute chest syndrome, cerebrovascular stroke, vaso-occlusive crisis, acute kidney injury, venous thromboembolism, and pulmonary embolism (PE). 5

The purpose of this article is twofold. First, the authors describe a case report with the successful use of free tissue transfer in a patient with SCD. Second, the authors perform a systematic literature review to identify previous cases of free tissue transfer in SCD, to determine published success and complication rates, and to review reported preoperative, intraoperative, and postoperative management strategies.

A retrospective chart review was performed of a patient with SCD who underwent free tissue transfer for soft tissue coverage of a large craniofacial defect. Medical history, preoperative workup and interventions, operative details, postoperative management, and complications were recorded. As a single case report, this study was exempted by the Institutional Review Board at our institution. Written consent was obtained for the use of patient photographs.

A systematic literature review using the Preferred Reporting Items for Systematic Reviews and Meta-Analyses guidelines was performed using the keywords “free tissue transfer,” “free flap,” or “microsurgery” and “sickle cell” on PubMed, Ovid/Medline, and Scopus. Any relevant publications identified in the references of these articles were also included for review. Two authors then independently evaluated these articles and identified those describing cases of free tissue transfer in SCD. Articles that described other types of flaps, described free tissue transfer in sickle cell trait (SCT), were about other topics, or were not written in English were excluded. The articles describing cases of free tissue transfer in SCD were queried for data on patient demographics, preoperative laboratory values, preoperative workup and interventions, indications for free tissue transfer, operative details, postoperative management, and complications.

## Case

**Fig. 1:**
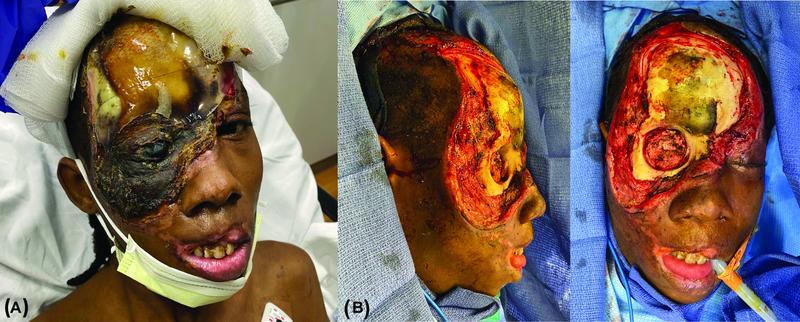
( **A** ) Patient appearance on emergency department presentation. There is a large right face and scalp eschar with overlying Integra silicone sheet still in place, and purulent drainage from the right globe. ( **B** ) Patient appearance after excisional debridement of face and scalp wounds and right orbital exenteration.

A 29-year-old African American male presented to the emergency department with right lower extremity swelling and a large, infected wound over the right face and scalp ( Fig. 1A ). Two months prior to presentation, he was pushed onto a subway live rail, suffering an electrical burn to the right face and scalp. He was admitted to an outside hospital, underwent excisional debridement and placement of Integra bilayer matrix wound dressing (Integra LifeSciences, Princeton, NJ), then left against medical advice, and was lost to follow-up. Past medical history was notable for SCD on hydroxyurea with prior episodes of vaso-occlusive crises and acute chest syndrome, history of deep vein thrombosis (DVT), cholecystitis status post-cholecystectomy, and chronic pain with opioid dependence. His state was further complicated by a complex social history including drug abuse and unstable housing.

The patient was admitted to our institution's Burn and Complex Wound Center. Notable admission laboratory findings included hemoglobin (Hgb) 7.8 g/dL and HbS 7.0%. Although his HbS value was unusually low, most likely due to receiving blood transfusions during recent admissions at outside hospitals, the patient's HbS 1 year prior was 55.3% which is more consistent with SCD. Venous duplex imaging demonstrated a partially occlusive DVT of the gastrocnemius vein corresponding to his right lower extremity swelling and pain. Hematology was consulted and recommended packed red blood cell (pRBC) transfusion with goal Hgb > 10 g/dL, exchange transfusion as needed to keep HbS < 30%, and 3 months of anticoagulation for the DVT. The patient was transfused 2 units of pRBCs to bring his Hgb to 10.0. As the HbS was 7.0%, no exchange transfusion was performed, and a therapeutic heparin drip was initiated for anticoagulation.

One day after admission, the patient underwent excisional debridement of the face and scalp wounds and right orbital exenteration, resulting in a large defect with exposed craniomaxillofacial bones ( Fig. 1B ). Intraoperative quantitative tissue cultures grew multiple drug resistant organisms including *Escherichia coli* , *Bacillus* , coagulase negative *Staphylococcus* , *Corynebacterium amycolatum* , and *Candida* which were treated with topical antimicrobials and intravenous broad-spectrum antibiotics per Infectious Disease recommendations.

**Fig. 2:**
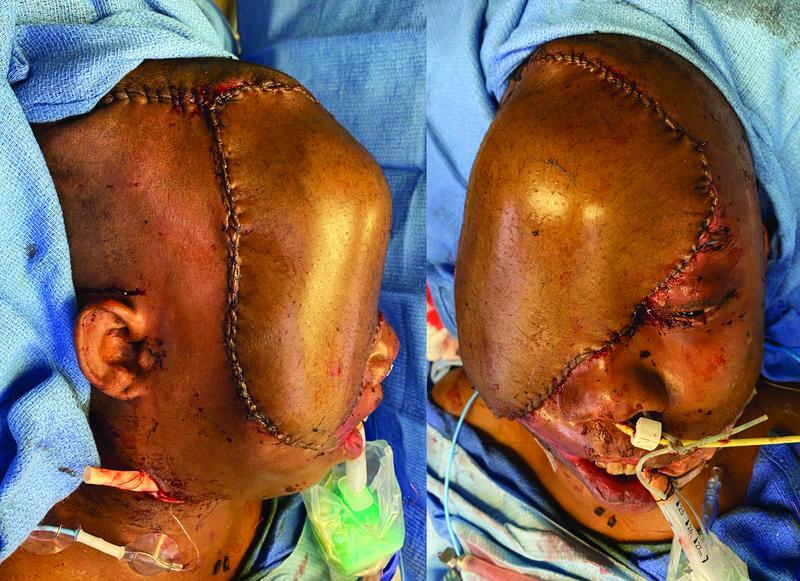
Immediate postoperative appearance after free anterolateral thigh (ALT) flap reconstruction.

One week after debridement, the patient underwent definitive wound closure with a left anterolateral thigh (ALT) free flap ( Fig. 2 ). Repeat HbS prior to reconstruction was 6.6%. The flap pedicle was anastomosed to the right superior thyroid artery and vein in end-to-end fashion. Technical issues requiring multiple revisions of the arterial anastomosis led to a total ischemia time of 133 minutes. Due to prolonged ischemia time, topical ice was used to cool the flap. Total operative time was 12 hours. Patient temperature was maintained between 36.1 and 38.1°C. He was transfused with 4 units of pRBCs and 2 units of fresh frozen plasma to maintain Hgb > 10 g/dL intraoperatively. The flap was monitored using a continuous implantable Doppler on the flap artery (Cook-Swartz Doppler Probe, Cook Medical, Bloomington, IN), continuous tissue oximetry monitoring (ViOptix, Newark, CA), cutaneous Doppler signals, and hourly clinical flap checks.

**Fig. 3:**
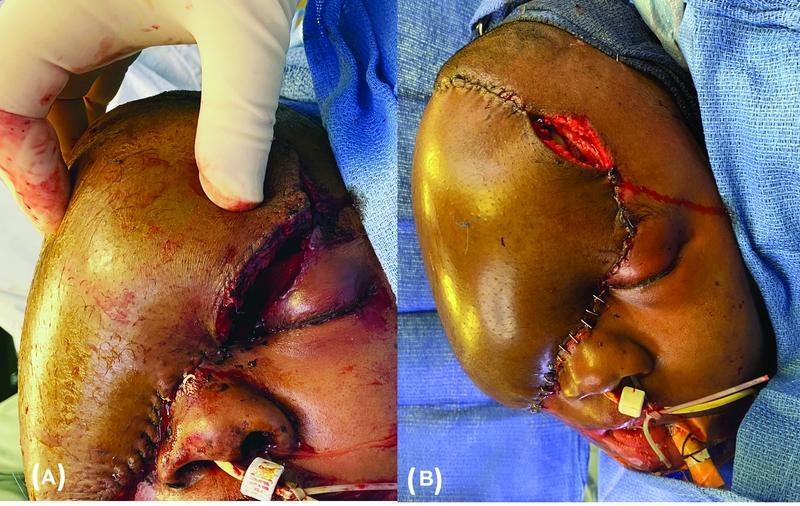
( **A** ) Right neck and flap swelling on postoperative day 1 causing dehiscence at the left upper eyelid suture line. Progressive swelling and downtrending ViOptix readings were concerning for hematoma causing impending flap compromise, and the patient was taken to the operative room urgently for flap exploration. ( **B** ) Flap re-inset after hematoma evacuation, leaving an open area at the left scalp.

On postoperative day 1, the patient developed progressive neck swelling at the site of the anastomosis, flap swelling causing dehiscence at the left upper eyelid suture line, and downtrending ViOptix readings ( Fig. 3A ). Operative exploration found old hematoma in the neck and under the flap but no active bleeding. The flap was unable to be reclosed due to swelling, so it was re-inset to allow for left upper eyelid closure, leaving a small part of the left upper scalp open ( Fig. 3B ). The hematoma was attributed to postoperative hypertension and supratherapeutic heparin drip (partial thromboplastin time > 100 seconds).

**Fig. 4:**
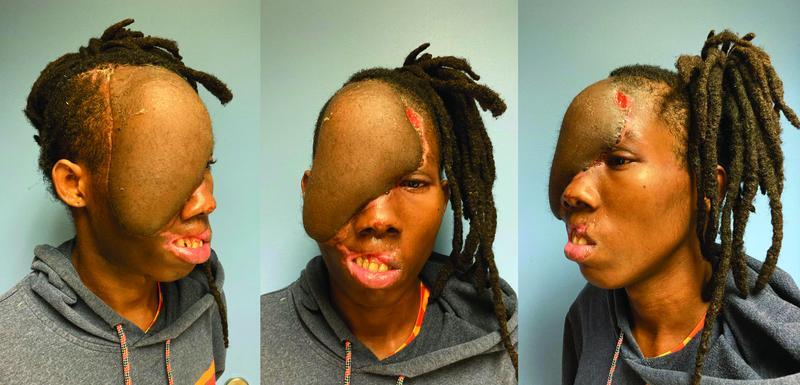
Patient appearance at 1 month postoperatively. The flap remains viable and the open wound on the left scalp is nearly healed with dressing changes.

The remaining hospital course was unremarkable from a flap perspective. The patient was transfused as needed to maintain Hgb > 10 g/dL. Postoperative HbS was < 5.0%. Anticoagulation consisted of aspirin 81 mg daily for empiric free flap thromboprophylaxis and apixaban 5 mg twice daily for 3 months for the right lower extremity DVT. There were no SCD-related complications. At 1-month follow-up, the flap remained viable and the left scalp wound was healing well with silver sulfadiazine dressing changes ( Fig. 4 ).

### Systematic Review

**Fig. 5:**
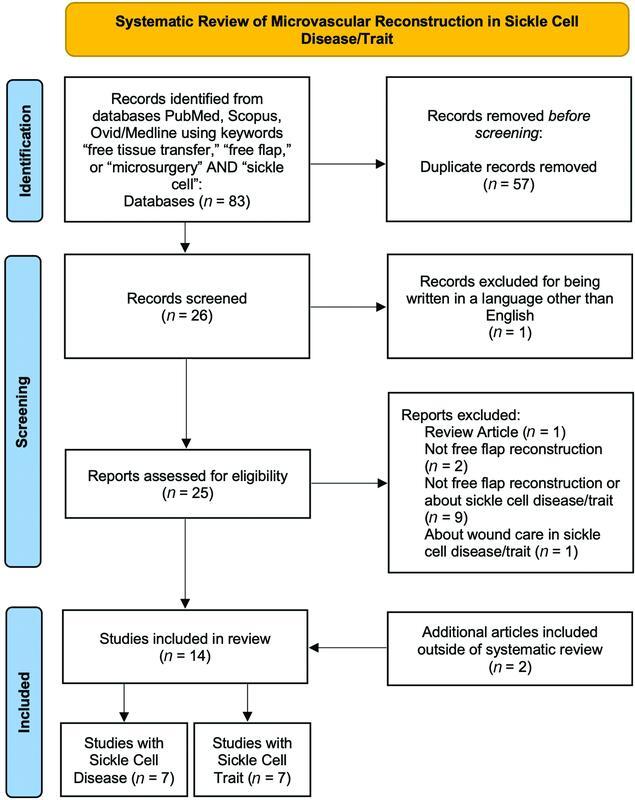
Preferred Reporting Items for Systematic Reviews and Meta-Analyses (PRISMA) diagram detailing systematic literature review using the keywords “free tissue transfer,” “free flap,” or “microsurgery” and “sickle cell” on PubMed, Ovid/Medline, and Scopus in September 2021. Articles that described pedicled flaps, that described free tissue transfer in sickle cell trait (SCT), were about other topics, or were not written in English were excluded. Seven articles describing cases of free tissue transfer in sickle cell disease (SCD) were identified.

As depicted in Fig. 5 , we identified 83 articles, 26 of which were unique and 57 of which were duplicates. We believe the smaller number of unique articles and larger number of duplicates reflect the paucity of reports on this topic. It also likely reflects that each database encompasses a vast majority of the available literature, leading to considerable overlap. Two additional papers were found outside of the literature search from references within these 26 identified articles. Of the total of 28 articles, 7 articles described 13 cases of free tissue transfer in 10 patients with SCD. 6 7 8 9 10 11 12 Preoperative, intraoperative, and postoperative details of each case are summarized in Tables 1 , 2 , 3 respectively. Seven articles describing free tissue transfer in SCT were excluded from analysis but reviewed in the “Discussion” section. 13 14 15 16 17 18 19

**Table 1:** Preoperative details of 13 free flap cases in 10 patients with SCD reported in the literature

Case number	Authors	Year	Age/gender	Hemoglobin genotype	Preoperative exchange transfusion	Preoperative or listed goal HbS (%)	Preoperative or listed goal Hgb (g/dL)/Hct (%)
1	Chang et al.	2022	37 M	HSBT	Yes	65 to 14	Hb > 10 g/dL
2	Weinzweig et al.	1997	36 F	HbSS	Yes	< 30	Hct 31–35
3	Weinzweig et al.	1997	36 F	HbSS	Yes	< 30	Hct 31–35
4	Weinzweig et al.	1997	35 M	HbSS	Yes	< 30	Hct 31–35
5	Weinzweig et al.	1997	35 M	HbSS	Yes	< 30	Hct 31–35
6	Monga et al.	1996	37 M	HbSS	Yes		
7	Weinzweig et al.	1995	25 F	HbSS	Yes	< 30	Hct 31–35
8	Weinzweig et al.	1995	21 F	HbSS	Yes	< 30	Hct 31–35
9	Weinzweig et al.	1995	38 F	HbSS	Yes	< 30	Hct 31–35
10	Richards et al.	1992	26 F	HbSS	Yes	74 to 23.6	12.6 g/dL
11	Khouri and Upton	1991	33 M	HbSS	Yes	Unknown to 24.0	
12	Khouri and Upton	1991	33 M	HbSS	Yes	26.0	
13	Spence	1985	19 M	HbSS	Yes	33.8	

Abbreviations: HbSS, sickle cell disease; Hct, hematocrit ; HSBT, hemoglobin S β-thalassemia.

**Table 2:** Intraoperative details of 13 free flap cases in 10 patients with SCD reported in the literature

Case number	Defect	Flap	STSG	Recipient vessels	Anastomosis technique	Ischemia time (min)	Operative time (h)	Additional operative details
1	Chronic osteomyelitis of the mandible	Adipofascial radial forearm	No	L facial artery and external jugular vein		16		No tourniquetNo cooling
2	R chronic leg wound	Hemi omentum	Yes	R posterior tibial artery and vein	Artery: end to side	90	12	Flap flushed with 200 mL of 5000 U heparin and dextran 40 solutionIV dextran 40 and ASA given prior to anastomosis
3	L chronic leg wound	Hemi omentum	Yes	L posterior tibial artery and vein		60	12	Flap flushed with 200 mL of 5000 U heparin and dextran 40 solutionIV dextran 40 and ASA given prior to anastomosis
4	R chronic leg wound	Hemi omentum	Yes	R anterior tibial artery and vein		90	11	Flap flushed with 200 mL of 5000 U heparin and dextran 40 solutionIV dextran 40 and ASA given prior to anastomosis
5	L chronic leg wound	Hemi omentum	Yes	L anterior tibial artery and vein		150	11	Flap flushed with 200 mL of 5000 U heparin and dextran 40 solutionIV dextran 40 and ASA given prior to anastomosis
6	Penile autoamputation	Radial forearm	No					Penile prosthetic device placed within neophallus
7	R chronic ankle wound	L latissimus dorsi	Yes	R posterior tibial artery and vein	Artery: end to sideVein: end to end with cephalic vein graft	50	7	Cephalic vein graft with two valves
8	L chronic ankle wound	R latissimus dorsi	Yes	L posterior tibial artery and vein	Artery: end to sideVein: end to end	90	7.5	None reported
9	L foot contact burn wound	R temporoparietal fascia	Yes	L dorsalis pedis artery and vein	Artery: end to endVein: end to end			None reported
10	L chronic ankle wound	L radial forearm		L posterior tibial artery and VC x 2	Artery: end to sideVein: end to end			None reported
11	L chronic leg wound	L latissimus dorsi	Yes	L dorsalis pedis artery and anterior tibial VC	Artery: end to endVein: end to end	16		None reported
12	R chronic leg wound	R latissimus dorsi	Yes	R posterior tibial artery and VC	Artery: end to sideVein: end to end			5000 U IV heparin bolus given at time of vascular clamping
13	L chronic ankle wound	Gracilis	Yes	L anterior tibial artery and vein		90		Flap cooling (20 minute of cold ischemia time)

Abbreviations: ASA, aspirin; IV, intravenous; SCD, sickle cell disease; STSG, split-thickness skin graft; VC, venae comitantes.

**Table 3:** Postoperative details and complications of 13 free flap cases in 10 patients with SCD reported in the literature

Case number	Postoperative Hgb goal	Postoperative anticoagulation	Flap compromise	Wound infection	STSG loss	Operative intervention	Flap survival	Wound coverage	Follow-up (mo)
1	> 8 g/dL		No	No	N/A	No	Yes	Yes	8
2	> 12 g/dL	IV dextran 40 × 5 dASA 325 mg × 2 wk	No	Yes (Pseudomonas)		Delayed skin grafting	Yes	Yes	24
3	> 12 g/dL	IV dextran 40 × 5 dASA 325 mg × 2 wk	No	Yes (Pseudomonas)		Delayed skin grafting	Yes	Yes	24
4	> 12 g/dL	IV dextran 40 × 5 dASA 325 mg × 2 wk	No	Yes (Pseudomonas)		Delayed skin grafting	Yes	Yes	
5	> 12 g/dL	IV dextran 40 × 5 dASA 325 mg × 2 wk	No	Yes (Pseudomonas)		Delayed skin grafting	Yes	Yes	
6			No	No		Delayed penile prosthesis revision	Yes	Yes	60
7			Yes (9 hours postop) - intraflap microvascular thrombosis	No		YesPOD0: flap explorationPOD4: flap debridement and re-insetPOD15: flap debridement and STSG	No	Yes	36
8			No	Yes (Pseudomonas)		YesPOD7: flap debridementPOD14: delayed skin grafting	Partial	Yes	17
9			No	No	Yes		Yes	Yes	55
10	Hgb > 12 g/dLHbS < 16%	None until complications, then IV heparin	Yes (3 d postop) - artery	No		YesPOD3: flap explorationPOD ?: flap debridement, skin grafting	No	Yes	
11		None until complications, then IV heparin and ASA x 10 days	Yes (2 d postop) - artery	No	Yes	YesPOD2: flap explorationPOD ?: delayed skin grafting	Yes	Yes	24
12		ASA x 2 wk	No	No	Yes	Delayed skin grafting	Yes	Yes	24
13			No	No	No	No	Yes	Yes	24

Abbreviations: ASA, aspirin; IV, intravenous; POD, postoperative day; SCD, sickle cell disease; STSG, split-thickness skin graft;.

Note: Flap survival - Flap survival was defined as “Yes” if there was complete flap survival, “Partial” if > 50% of the flap survived, and “No” if < 50% of the flap survived.

Patient ages ranged from 19 to 38 years old (average 30.7 years), with five males and five females. All cases had preoperative exchange transfusions. Goal HbS was < 30%, except for one case where it was 33.8% and one where it was not reported. All cases had a Hgb transfusion goal of > 10 g/dL, except for four cases where it was not reported ( Table 1 ).

Free tissue transfer was indicated for lower extremity wounds ( *n* = 11), chronic mandibular osteomyelitis ( *n* = 1), and penile autoamputation secondary to recurrent ischemic priapism ( *n* = 1). Free flaps included latissimus dorsi ( *n* = 4), hemi omentum ( *n* = 4), radial forearm ( *n* = 3), temporoparietal fascia ( *n* = 1), and gracilis ( *n* = 1). Ischemia time ranged from 16 to 150 minutes (average 72 minutes) and was not reported for four cases. Total operative time ranged from 7 to 12 hours (average 10.1 hours) and was not reported for seven cases. Four flaps were flushed with a 200-mL solution consisting of 5,000 units of heparin and dextran 40 solution prior to anastomosis. Five cases reported systemic anticoagulation prior to anastomosis, four cases gave intravenous dextran 40 and enteral aspirin, and one case gave a bolus of intravenous heparin 5,000 units ( Table 2 ).

The most common postoperative complications were flap compromise in three flaps and superficial wound infection in five flaps. These cases of superficial wound infection resulted in partial skin graft loss requiring repeat skin grafting in delayed fashion. However, no cases of superficial wound infection led to flap loss. Flap compromise was caused by arterial thrombosis in two cases and intraflap microvascular thrombosis in one case. Postoperative anticoagulation trends were variable. Four cases used intravenous dextran 40 for 5 days and aspirin 325 mg daily for 2 weeks. One case reported the use of aspirin for 2 weeks but did not specify the dose. Two cases did not use anticoagulation until signs of flap compromise. Ten out of 13 flaps required further operative intervention with flap exploration ( *n* = 3), flap debridement ( *n* = 3), repeat skin grafting ( *n* = 9), and penile prosthesis revision ( *n* = 1). Ten cases reported complete flap survival, one reported survival of more than two-thirds of the flap, and two reported loss of more than half of the flap. However, all defects were successfully closed with follow-up of 8 to 60 months ( Table 3 ).

Flap compromise occurred in cases 7, 10, and 11. In case 7, flap compromise was noted 9 hours postoperatively with decreased cutaneous Doppler signal and poor flap bleeding after a transient hypothermic and hypotensive episode. 9 Operative exploration showed patent pedicle vessels, and flap compromise was attributed to microvascular thrombi in the flap circulation. The flap was managed expectantly with two further debridements removing 75% of the flap thickness, but the wound was successfully closed with skin grafting. No information on postoperative Hgb, HbS, or anticoagulation was reported.

Flap compromise in case 10 was noted on postoperative day 3 with a pale flap appearance. 10 Operative exploration showed extensive thrombus in the posterior tibial artery, starting in the proximal lower leg and extending into the flap. Flap salvage was attempted with Fogarty catheter thrombectomies, a bolus of intravenous heparin 5,000 units, revision of the arterial anastomosis from end-to-side to end-to-end, and flap infusion of 35,000 units of streptokinase for 1 hour. Ischemia time was 6 hours and most of the flap demarcated. Interestingly, histology of the debrided flap showed thrombus without sickled cells, and HbS at the time of flap compromise was 16.6%. The wound was successfully closed with skin grafting.

Flap compromise in case 11 was noted on postoperative day 2 with decreased flap temperature. 11 Operative exploration showed thrombus at the arterial anastomosis extending into the flap. Flap salvage was successful with flap infusion of streptokinase and anastomosis revision from end-to-end to the dorsalis pedis artery to end-to-side to the anterior tibial artery. Ischemia time was 4 hours. Systemic anticoagulation was also instituted with intravenous heparin and aspirin for 10 days. Postoperative HbS was not reported.

## Discussion

SCD has traditionally been considered a contraindication for free tissue transfer due to concerns of higher risks of flap failure from sickling and generalized hypercoagulability. This article reports a successful case of a free ALT flap in a patient with SCD and presents a systematic literature review identifying 13 cases of free tissue transfer in 10 patients with SCD.

All 13 cases used exchange transfusions and/or pRBC transfusions preoperatively, with most cases reporting goals of lowering HbS to < 30% and raising Hgb to > 10 g/dL. The concept of using preoperative blood transfusions in SCD to elevate hemoglobin, reduce reticulocyte count, and suppress sickle cell production was first described in 1969 for patients undergoing skin grafting for sickle cell ulcers. 20 The idea of using preoperative exchange transfusions emerged later, as it was found that the filterability of blood in SCD patients is normal when HbS is < 40% and that vaso-occlusive crises occur only when HbS is > 50%. 21 22 A 2020 Cochrane meta-analysis found insufficient evidence to recommend aggressive preoperative blood transfusions in SCD to prevent sickle-related or surgery-related complications, 23 but it should be strongly considered in free tissue transfer where the risk of sickling and thrombosis should be minimized.

While the main focus of preoperative optimization is on the hematologic system, it is worth mentioning that SCD affects all organ systems, especially the respiratory, gastrointestinal, neurologic, and immunologic systems. 5 Our patient was relatively young but had many of the chronic manifestations of SCD, like all cases in the literature review with an average age of 30.7 years. This is likely due to selection bias, as older SCD patients may be considered poor free flap candidates in an already high-risk population. In addition, the projected life expectancy for people with SCD versus general population in the United States is 54 versus 76 years. 24

Our review did not identify any operative considerations to minimize the risk of flap loss. Because sickling is induced by local tissue hypoxia, cold temperatures, and circulatory stasis, it is reasonable to assume that flap ischemia time, flap cooling, and tourniquet use should be minimized. However, the three cases that suffered thrombotic events had reported 50 and 16 minutes of ischemia time (not reported for case 10). Our flap ischemia time was 133 minutes, and we cooled the flap due to longer ischemia time. The decision to use topical ice to cool the flap was made in this case because the authors believed that the risks of prolonged warm ischemia time causing irreversible tissue death outweighed the risks of cold temperature causing sickling. Upon review of the available cases, case 13 was also cooled for 20 minutes and completely survived as well. 12 McAnneny et al reported a case of bilateral immediate transverse rectus abdominis muscle flaps in a patient with SCT who suffered unilateral flap thrombosis and failure on the side that was cooled in an ice bath for 25 minutes. 14 Pathology showed sickled cells in the flap microvasculature. Because the two flaps were otherwise treated the same, the authors concluded that flap cooling was the principal cause of intraflap microvascular thrombosis. In our review, a tourniquet was not used in one radial forearm free flap and was not reported in the two other radial forearm free flap cases, making it difficult to draw conclusions about whether tourniquet use during flap elevation affects flap survival. 6 8 10 Two literature reviews report that tourniquets can be used safely in patients with SCD and SCT, but with a complication rate of 12.5% including extremity swelling, severe pain, infection, or DVT/PE. 25 26

The reported use of intraoperative intraflap and/or systemic anticoagulation was mixed. In their 1997 case series, Weinzweig et al flushed their flaps with 200 mL of dextran 40 and 5,000 units of heparin and administered systemic anticoagulation with intravenous dextran 40 and aspirin prior to anastomosis, 7 but the same group did not report using this protocol in their 1995 case series. 9 Neither article reports why these changes were made. Intraflap and systemic anticoagulation were used to help rescue cases 10 and 11 during operative takeback for flap arterial compromise.

Our review also did not identify any recommendations on postoperative management. Again, it is reasonable to assume that triggers of sickling should be avoided, and thus patient normothermia, oxygenation, hydration, and analgesia should be maintained. In addition, close monitoring of pulmonary, gastrointestinal, renal, or thromboembolic dysfunction is required as SCD patients are at an estimated sickle-related postoperative complication rate of 17% with mortality figures of 1%. 5

There were three reported cases of flap compromise caused by arterial thrombosis in two cases and intraflap microvascular thrombosis in one case, 9 10 11 despite preoperative optimization to lower HbS and raise Hgb ( Table 3 ). Of note, the timing of arterial thrombosis was relatively delayed in cases 10 and 11. Although preoperative exchange transfusions and pRBC transfusions dilute abnormal erythrocytes, SCD patients still have generalized hypercoagulability. This suggests that postoperative anticoagulation should be considered.

Postoperative anticoagulation protocols from our review were varied ( Table 3 ). In our case report, we initiated a therapeutic intravenous heparin drip preoperatively for DVT treatment and added aspirin 81 mg daily postoperatively for flap thromboprophylaxis. However, the use of both intravenous heparin and aspirin likely contributed to our patient's hematoma, causing acute flap compromise. Similarly, Young-Afat et al report a patient with SCT who was treated with therapeutic low molecular weight heparin after a unilateral deep inferior epigastric perforator flap complicated by hematomas at both the flap and donor site requiring operative takeback. 16 These examples highlight the fine balance between managing complications from bleeding versus clotting in this unique patient population.

The second most common postoperative complication in our systematic review was superficial wound infection in five patients causing skin graft loss. Interestingly, all reported infections were caused by *Pseudomonas* ( Table 3 ). Studies on the microbial flora of sickle cell chronic leg wounds have shown that *Pseudomonas aeruginosa* is the second most common bacteria isolated after *Staphylococcus aureus* . 27 28 This likely reflects differences in microbial flora in SCD patients due to their functional asplenia and relative immunocompromise, making them at higher risk for infection with encapsulated bacteria. 29

Although only 10 out of 13 flaps completely survived, all defects were successfully reconstructed. Even in cases of > 50% flap loss, enough of the flap survived to allow for skin grafting over a vascularized wound bed. The concept that “free tissue failure is not an all-or-none phenomenon” is an important one, 30 particularly in a patient population that is traditionally considered not to be good candidates for free tissue transfer.

This article describes a case report and systematic literature review on 13 cases of successful free tissue transfer in SCD. Due to the paucity of cases, it is difficult to draw specific conclusions on how to best approach free tissue transfer in SCD. All cases recommended preoperative optimization by reducing HbS to < 30% and keeping Hgb > 10 g/dL to minimize sickling and reticulocyte production of abnormal cells. There were no generalizable trends regarding intraoperative or postoperative optimization. It is important to be mindful that the pathologic effects of chronic hemolysis, microvascular thrombosis, and vaso-occlusive events can lead to progressive end-organ damage as well as a proinflammatory and hypercoagulable state, and that these effects are systemic and require multidisciplinary care. For complex defects in SCD patients, reconstruction with free tissue transfer can be successful with medical and surgical optimization.
